# SSRI antidepressants and perceived loss of lean muscle in men: A qualitative exploration of some online anecdotal concerns

**DOI:** 10.1177/09246479251346445

**Published:** 2025-05-23

**Authors:** Nicholas Norman Adams

**Affiliations:** 1 The School of Health, 1018 Robert Gordon University, Aberdeen City, Scotland

**Keywords:** SSRI antidepressants, SSRI side-effects, lean muscle loss, social pharmacy approaches

## Abstract

**Background:**

This study examines anecdotal reports from online discussion forums suggesting possible links between SSRI antidepressants and loss of lean muscle mass, particularly in men. Given limited existing scientific research, this study bolsters academic discourse.

**Objective:**

Do self-reported experiences from internet forums indicate a perceived connection between SSRI use and muscle mass reductions?

**Method:**

A Google keyword search identified 202 posts from 14 randomly selected online antidepressant discussion forums. Posts were collected and thematically analysed.

**Results:**

Forum users reported difficulties in maintaining or gaining lean muscle after commencing SSRI treatment. Key themes included frustration, confusion, and attempts to rationalise perceived changes.

**Conclusion:**

Findings suggest an area for further exploration, regarding the physiological impact of SSRIs on muscle composition. While reports remain anecdotal, they highlight concerns immediately relevant to both patients and healthcare professionals. As the study is based on self-reported experiences from anonymous sources, findings lack scientific validation but highlight requirements for further studies to explore prevalence and broader applicability. Research observations spotlight a need for further, structured clinical research to investigate possible effects of SSRIs on muscle mass.

Future research should include controlled clinical trials and longitudinal studies to examine a potential association in more detail.

## Introduction

Antidepressants within the Selective Serotonin Reuptake Inhibitor (SSRI) family are some of the most widely prescribed medications in the world.^1,2^ Use of SSRIs as longitudinal front-line treatments for major depressive disorder (MDD), anxiety, and social phobias among other mental illnesses have allowed for significant research on tolerability and side-effects to be developed.^3–5^ While side-effects such as sexual dysfunction (SD),^6^ apathy,^7^ and suicidal ideation,^8^ and more recently post-SSRI sexual dysfunction (PSSD),^9–11^ have been studied, there exists only brief scholarly literature examining linkages between SSRI use and perceived physical reductions in lean muscle mass or difficulty building muscle mass. This is despite a body of publicly available anecdotal self-reported patient-experiences that exist within online discussion forums that uphold a perceived connection.

In contemporary society, interest in social pharmacy approaches as a method of understanding patient-experiences is rapidly growing,^12^ particularly with regards to gathering qualitative data exploring patients’ lived experiences of antidepressant use and side-effects.^3,13,14^ Many scholars now uphold the value of considering sociological investigations anchored in ‘real world’ patient experience as a valid adjunct to the traditional quantitative metrics that inform clinical trials.^3,15–18^ Such qualitative, anecdotal patient data holds value not only as a complement to clinical study outcomes reporting ill-effects, but also as knowledge to inform prescribing policy for ‘real world’ medical practice. In this context, this research study serves to highlight from a qualitative, self-report, sociological perspective some relevant experiences of persons taking antidepressants, including SSRI class medications.

The research aim is that by drawing attention to self-reported discussions highlighting a possible user-perceived connection between SSRIs and lean muscle loss, further scientific inquiry may be encouraged that actively investigates subjective patient-experiences of physical changes, and attempts to generate further research investigation on this matter. Research may seek to ascertain if there is an SSRI-mediated biological pathway that underpins such a side-effect, or alternatively, dispel such a connection through study of individuals perceiving such effects. Subsequently, new research may encourage prescribers to consider, where clinically appropriate, different-class medications for patients already suffering pre-existing muscle deterioration, physically weakening conditions, or anxieties and negative perceptions surrounding physical appearance.

## Methods

This study explores online discussions concerning a potential correlation between SSRI antidepressant use and self-reported loss of lean muscle mass. Using a qualitative online ethnographic approach, the research examines publicly available discourse to understand subjective experiences and patterns of concern. A thematic analysis, informed by Braun and Clarke’s six-phase methodology, was employed to systematically identify and interpret key themes within user-generated accounts. Positioned within an interpretivist qualitative paradigm, the study seeks to illuminate how individuals construct meaning and explanation around SSRI-related physical changes within digital peer communities.

Data collection was conducted via structured searches using Google (Advanced) with the keywords ‘antidepressants’, ‘muscle loss’, [and] ‘side-effects’. Fourteen publicly accessible online forums containing a total of 202 discussion posts were selected based on relevance and availability. These posts were imported into NVivo for qualitative coding, with iterative thematic categorisation used to identify emergent patterns. Key coding dimensions included discourse surrounding antidepressant usage, perceptions of physical transformation, and explicit references to perceived changes in muscle composition. The dataset was further stratified by gender, and where possible, associations were drawn between reported experiences and specific SSRI medications (if this information was available).

Ethical considerations were integral to the research design. Given that all data were sourced from publicly accessible online forums, ethical guidance from the British Sociological Association (BSA) and the British Psychological Society (BPS) was followed to ensure responsible data handling. A summary of this approach is discussed in the section below. Direct engagement with forum users was avoided, and no private or restricted content was accessed. To mitigate risks of re-identification, all quoted material was paraphrased, preserving semantic accuracy while preventing the possibility of reverse-searching original sources.

Written online accounts, as publicly available forum postings, were downloaded and imported into the qualitative analytical software program NVivo. Thematic Analysis was used to code linkage themes within data. This was carried out as inspired by Braun and Clark’s six-stage methodology which has seen success coding both complex, and simple qualitative data drawn from comparable online sources.^19–21^ Firstly, initial ‘start’ themes were established in three review passes of the imported data: data were coded for emergent themes including discussions of antidepressant medication use, reasons for use, and discussions of perceived medication effects and side-effects. Data were then separated into correlative case themes. Saliently, language dictating concerns surrounding antidepressant side-effects, language linking to concerns for physical changes, and language highlighting reductions in lean muscle mass were categorised. Thematic data was then re-coded by gender, then reviewed in a penultimate pass for prevalence of passages where subjects explicitly linked their antidepressant use to their perceptions of physical changes. A final additional level of coding was used to link descriptive comments to specific types of antidepressant – where this information was available. Most notably for men, some posts expressed concerns for a correlation between beginning SSRI class antidepressant medications and concurrent self-perceived loss of lean muscle mass.

### Ethics involving online-based research

The online data gathered to conduct this study lies entirely in the public domain, is freely available using any web browser and/or internet search engine, and the researcher had no contact with any posters of data. While considering the use of data from publicly available forums, both the British Sociological Association (BSA) guidelines on the research use of publicly available online forum data,^22^ and the British Psychological Society (BPS) ethics guidelines for internet-mediated research^23^ were consulted. Presented in the BSA advice, Eysenback and Till suggest ‘…it is ethical to record activities in a public place without consent, provided individuals are not identifiable. Human subject research norms such as informed consent do not apply to material that is published’.^22(p. 2)^ However, as Bruckman (2004) points out, the complex specifics of online content render it difficult to clarify concretely the concept of publication.^22(p. 6)^ Similarly, the BPS guidelines state:

Where it is reasonable to argue that there is likely no perception and/or expectation of privacy [for example, in online, publically available forums] […] use of research data without gaining valid consent may be justifiable. However, particular care should be taken in ensuring that any data which may be made accessible as part of the research remains confidential […] often achieved by ensuring anonymity […].^23(p. 8)^

At the time of developing this project, the data used was publicly available online. Data-availability was cross-checked when finalising data analysis. However, I have remained mindful that the rights of forum posters allow such users to later remove their online posted comments if they wish, without assuming their verbatim comments may also be held at an alternative online repository or print locale (i.e. within a linked data research repository or in a published research paper, such as this one).

Equally, I consider that while forum posters recognise the public availability of their posted data, they likely did not intend this data to comprise a component of academic research. Related to this point, there is a concerning trend within some online-focussed sociological research for scholars to present online forum quotations verbatim and in full, without consideration for the possibility that simple reverse-searching can often readily reveal the origins of such postings, and in some cases the identity of original posters, their usernames (or real names), and locational and personal data.^24^ The practice of using verbatim quotations is supported to some degree in the BSA^25^ literature by a quote from Bruckman (2004): ‘For web data […] personal details should be disguised; however, quotes may be used to evidence any findings’.^6,22^ Nevertheless, this thinking does not acknowledge the above confidentiality issues presented by using verbatim quotations from online forums.

Considering the above arguments and guidelines collectively, this research has taken significant steps to maximise anonymity for all data used. This has been achieved by rewording quotations to prevent discovery of these via reverse-searching. All quotations edited for rewording retain **any and all** original meaning. This protection maximises anonymity of any publicly available materials used in this research. Prior to submitting this manuscript for consideration, all edited quotations used were reverse-searched extensively, using both Google and Google Advanced searches, using both isolated and complete fragments of text. At the time of submission for publication, none of the edited quotations led back to the original online research materials and original forum postings.

## Results

A total of 202 postings were downloaded and analysed, drawn from 14 online forum groups. Almost all online postings were by men. Only two discussion posts were from women. A process of (non-reworded) quotation cross-checking was completed to ascertain if data drawn from forums remained ‘live’ and online at the time of analysis completion. All data remained traceable and located online at this time. During this process, some additional forums containing similarly focussed postings were discovered. However, these were excluded to avoid cherry-picking new forums with desirable quotations that could bolster (i.e. bias by inflating) the initially selected sample. However, notable of these forums was that some posters – also perceiving themselves to be experiencing SSRI-related muscle loss, expressed concerns that there is little published information or research available on the subject of a SSRI-lean-muscle-loss link. Within the analysed sample of 202 postings, three interlinked themes were present. These are discussed below.

Thematic analysis identified three notable areas of interest. First, a substantial number of forum users reported perceiving unexplained physical changes, with particular emphasis on reductions in lean muscle mass following SSRI initiation. Second, some participants expressed frustration at the perceived lack of medical recognition, noting that healthcare professionals frequently dismissed their concerns. Third, online forums functioned as key sites of peer support, where individuals exchanged experiential knowledge, personal coping strategies, and alternative explanations for their symptoms. These themes are discussed in detail below.

### Theme 1: SSRIs and recovery from depression

The primary topics of discussion forums pertained to side-effects; therefore, positive discussions of SSRI antidepressants were rare in forums. However, acknowledgement of SSRI antidepressant-mediated relief from depression, and positive mental effects of SSRI medications were mentioned in some cases. Some posters acknowledged that their self-perceived levels of anxiety, as well as depression had decreased since starting SSRI medications: ‘After being prescribed Lexapro (Escitalopram), I noticed a significant improvement after 3 weeks. My depression and anxiety have greatly diminished since starting the medication’/‘I’ve noticed a positive change in my anxiety since beginning the SSRI’/‘I feel more at ease since starting the SSRI’. Anecdotal findings are congruent with the large body of clinical literature reporting SSRI medications as powerful and essential front-line agents for successfully treating MDD.^2,3,7,14,26^

### Theme 2: Discussion of bothersome side-effects of medication(s)

Despite the above theme of recovery, posters also often spoke of negative side-effects linked to SSRI class medications. For some, this included well publicised sexual side-effects such as inability to achieve orgasm^6,9,13,14^ and sex drive being ‘noticeably lower’. Others spoke of ‘tingles’ or feeling dizzy when missing prescribed doses for one or several days. Still others discussed side-effects of ‘mood swings’ and ‘feeling very angry and irritable’, effects to appetite (both increase and decrease were noted), and reduced motivations: ‘my motivation is absent’/‘I have zero motivation for anything’. Such effects are all well noted in existing literatures.^3,5,9,15^

### Theme 3: SSRIs and ‘loss of lean muscle mass’

Most significantly, postings present within the 14 forums addressed the topic of ‘losing lean muscle’ and perceived reductions in muscle mass and muscle tone, since beginning SSRI medications. Almost all postings discussing this topic were from men, except for two, posted by women. Most interestingly, some postings described near-identical presentations.

For example, one male: ‘R’^1^ spoke of beginning an SSRI medication (medication name not specified) to treat depression and anxiety. After a short spell on medication, his mood began to lift. However, he voiced that these improvements coincided with a shift in body composition, describing a perceived rapid loss of muscle mass and a noticeable increase in body fat, with loss of definition in all muscles (most notable in the stomach, chest, and arms), and the self-identified onset of gynaecomastia. He stated:

‘I noticed that the make-up of my body began to change quickly. I began to shed my naturally lean muscle and I grew a layer of fat, which gave me a “man boob” look. To me it seemed like all the muscle in my body got soft, significantly, within a matter of weeks’

‘R’ perceived changes to occur despite ‘R’ voicing that no modifications had been made to his existing diet and exercise routine, which ‘R’ stated been established prior to beginning medication. ‘R’ recounted discontinued medication, citing concerns over ‘the medication changing testosterone levels’ but was anxious to note that muscle loss side-effects remained: ‘despite engaging in ongoing exercise, lifting weights and eating healthy – the problem has not resolved’. ‘R’ concluded that these physical changes significantly impacted his mood in the negative, to a degree worse than his previously depressed state, which he originally sought medical help for:

‘I’m really worried about what has happened to my body (mostly with my chest). I feel even worse than that way I did before beginning the medication. I am worried that my body’s composition may have now permanently shifted, and I don’t know what else to do now’

Several other males, replying in the same forum shared similar stories of muscle tone and bodily composition changes while taking ‘antidepressants’. However, these postings did not explicitly specify that the antidepressant medication was an SSRI class drug.

One man: ‘C’ identified as having the ‘same problem’ as ‘R’. He cited an increase in fatigue, body fat, and ‘man boobs’ despite describing reasonable diet and exercise. Changes were described in the context of physical recomposition, hallmarked by redistribution of body fat, reduction of lean muscle mass, and perception of noticeable and newly formed (self-identified) gynaecomastia:

‘I felt tired all day every day. My arms became less defined as the tone of the muscle began to become reduced – despite the fat located around my stomach not really stressing me, the “man breasts” do stress me’

Like ‘R’ this poster expressed concern regarding the permanence of these effects, attributing occurrence to the antidepressant medication:

‘The “man boobs” appeared while taking the antidepressant medication. I’ve been stopped the medication for three months now but the side-effects are still there to a degree – mainly the fatigue I’ve been experiencing’

Another poster: ‘M’ commented similarly, again in the same discussion thread. Like ‘R’ and ‘C’ his concerns echoed the themes of reductions in muscle loss and bodily recomposition: ‘The same thing happened to me…’. ‘M’ discussed that while he had maintained his workout intensity and ‘in fact ramped-up the intensity of workouts in the last four months’, since beginning an antidepressant, his ‘body composition has shifted, and [he was] worried’. Notably, ‘M’ highlighted the impact of these physical changes as compounding his negative mental state: ‘Self-image is a significant factor in my low mood; my depression – I am taking antidepressants’. Reflecting on specific changes, he again highlighted identical presentations to that of ‘R’ and ‘C’:

‘[I’ve lost muscle] throughout my chest and abdomen. I can’t gain any more muscle – since I began the medication it’s been different; my weight has stayed the same but absolutely I’ve lost muscle tone’

‘M’ concluded by speculating that the medication affected his energy levels, and that perhaps this was a factor in the bodily changes he perceived to be experiencing.

On a different forum, another male poster: ‘N’ discussed his experience of ‘antidepressants’. He commented that he had been weight training for over 7 years, and maintaining serious, dedicated training for the past 4 years. In this time, he discussed how he had made ‘regular, ongoing, strength and muscle gains’. However, since beginning an antidepressant he noted: ‘Since beginning an antidepressant, I’ve gone backwards in both strength and physical appearance’. He queried a correlation: ‘Is there any correlation?’, suggesting that it seemed ‘weird’ that despite ‘eating the same number of calories’ and ‘working out just as vigorously, if not more’ that he should experience such physical changes only after starting an antidepressant medication.

Posters on other forums shared comparable stories to the above; this time, highlighting specific links to SSRI class antidepressants. One poster: ‘C’, commented that since starting an SSRI (Citalopram), his ability to workout had decreased, his ‘ability to lift weights and do running has gone down, and muscle mass has rather declined’. ‘C’ commented that he was unable to find much information about this ‘on the internet’ and asked other forum posters whether SSRIs were the cause, if other information was available, and whether there were alternative medications without these effects.

Another poster: ‘H’, taking the SSRI Paroxetine, voiced that he had made several internet threads seeking information about a possible correlation between SSRI antidepressants and muscle loss, but could find little available information. He posted that since beginning SSRI medication ‘The medication is making me lose strength’, suggesting he felt his SSRI medication had ‘definitely reduced testosterone levels’. He bookended these comments by offering personal anecdotal examples of muscle weakness and reductions in strength, specifically, losing informal strength-based competitions to friends who did little exercise. He claimed that despite working out, he still felt weak and had little muscular strength compared to his pre-SSRI baseline: ‘Overall, it feels now like I have a much more difficult time gaining strength, reducing weight and building physical definition and muscle’.

Another poster in a forum discussion surrounding SSRI medications and rapid loss of muscle mass was ‘T’. ‘T’ discussed how he had been bodybuilding for 4 years. He was now in his late 20s, and had taken the SSRI antidepressant Prozac (Fluoxetine). Surprisingly, and mimicking the presentation of other posters on different forums, he also spoke of muscular changes in specific areas of his body – his chest. He stated:

‘While taking the antidepressant, I noticed a significant change in the shape of my chest. It no longer looks as it did before – I used to have a naturally well-developed chest with great shape, which was something I took pride in physically. Despite my efforts in the gym, I haven’t been able to restore it to its former appearance. This has made me feel very self-conscious and has caused me to lose all motivation for exercising’

‘T’ recounted how this made him feel psychologically: ‘I’ve been so frustrated – more than I have been in my life up until now. I have strived hard to build my body, it’s so disappointing to see it vanish for no apparent reason’. He continued by querying whether taking the SSRI medication could have ‘triggered something to shift my muscle fibres permanently’. He concluded: ‘I’m worried I might have really messed-up my body [by taking the SSRI] and am freaking-out’.

A poster on a different forum, with a different username: ‘J’, spoke also of links between beginning Prozac (Fluoxetine) and reductions in lean muscle mass, again – specific to his chest area. ‘J’ spoke first of beginning the SNRI medication Cymbalta (Duloxetine), before ‘moving over’ to the SSRI medication ‘Prozac’. He stated: ‘I was on Prozac for about 1 month then stopped as I did not feel it working’. ‘J’ spoke of how ‘within that month’ he had ‘relaxed going to the gym for around 2 weeks’ and was surprised to discover that ‘all [his] muscle mass had vanished – especially in the inner and upper chest’. He stated that he had since returned to the gym and had been training intensely for around a month, yet had seen no perceived improvements: ‘I’ve not noticed an improvement at all, my chest is lacking and my shoulders aren’t as wide as they used to be’. He concluded that he was finding the situation highly stressful: ‘This reduced image of my body that I now see is contributing a tremendous level of stress to my depression’.

One of the only two female posters identified of the total sample was taking a Serotonin-Norepinephrine Reuptake Inhibitor medication (SNRI). SNRIs are similar to SSRI class medications, but also inhibit the reuptake of norepinephrine, in addition to serotonin. The poster: ‘F’, shared similar muscle loss experiences to the men within the SSRI and non-specific medications samples. ‘F’ recounted how after beginning an SNRI medication she had ‘Seen [that she was] becoming softer and weaker 1 year into [SNRI therapy]’. She stated that the realisation of this was ‘alarming’ due to her role in an employment position that required physical strength. She went on to discuss: ‘it was my physio that first noticed the weakness, first in my legs – the reduction in muscle tone’.

### Summary

Some forum posters upheld that since beginning SSRI (and in one case SNRI) class antidepressants they had gained fat and lost muscle mass and tone, and struggled to build new muscle mass. Others spoke of maintaining or even increasing their workout intensity (i.e. both weight lifting and cardiovascular exercise) but still noticed body changes concerning seemingly perceived rapid reductions in lean muscle. Some spoke of how self-image was an initial factor in their depression, and such noticeable subjective loss of lean muscle was having a negative effect on their mental health, self-esteem, and self-assessed quality of life. Posters spoke of feeling ‘soft’, ‘[muscles losing] their natural tone’/‘muscle mass decreasing’, and perceived reductions in muscle mass in specific areas, with several posters notably discussing specific loss in the chest area, as well as shoulders and ‘abs’ – abdominal areas. Most concerning of some anecdotal conversations was the shared acknowledgement that since some posters had discontinued medications, they felt perceived loss of lean muscle had not resolved, and some men recounted still struggling to build lean muscle, where these posters previously reported gaining muscle easily. Almost all posters attributed this to their antidepressant use. Some posters discussed how medications had ‘impacted negatively upon their daily lives’ among other negative comments: ‘When I was taking SSRIs I felt quite removed from life; I had little motivation to do anything’/‘I would suggest people are cautious about taking an SSRI if you are not already taking one’.

Significant is that several postings within the forums analysed contained speculation from men of the possible mechanisms of action that could underpin a perceived link between SSRI medications and loss of lean muscle mass. One poster: ‘DC’ speculated on a connection between beginning SSRI medications and links to lowering testosterone. This was a prevalent topic of debate present in several forum conversations, and some men shared that they perceived a connection between SSRI medications causing lowered testosterone levels. At times, these discussions also linked back to the perceived prevalence of muscle loss and fat gain in the chest area, with some posters highlighting a perceived link between SSRIs, lowered testosterone levels and gynaecomastia. ‘DC’ commented:

‘I once read somewhere about SSRIs messing with test [testosterone] levels – I’ve been on Sertraline (discontinued taking), Lexapro [Escitalopram] – which I’ve stopped taking, and Prozac – which I’m getting ready to stop taking now’

‘DC’s comments suggest that some posters felt that their concern over SSRI-mediated changes in testosterone were sufficient for them to discontinue their medications. This perception was shared by others. One poster, ‘M’ commented: ‘I heard SSRIs can minimise testosterone production too’. Referring to weight-trainers using SSRIs, he posted: ‘I have heard some guys will use SSRIs to help with recovery, after training […] This might have been prior to it being more widely known that SSRIs can reduce T [testosterone] levels’.

Other posters on same and different forums commented with similar themes. ‘AN’ commented: ‘Antidepressants, especially SSRIs drop-down testosterone and increase oestrogen’. Another poster: ‘AR’ commented ‘SSRIs absolutely increase oestrogen in the body and they have been known to cause gynecomastia’. Others provided further comments, ‘TU’ commented: ‘I’ve been on SSRIs while also exercising, and there may be a link between low testosterone (in males) and SSRIs in my opinion’. ‘RT’ commented: ‘SSRIs increase oxidation within testicles and this adversely impacts the production of testosterone’. ‘UM’ commented: ‘For me, I’ve heard SSRIs like Paxil [Paroxetine] can dampen-down testosterone levels and make it harder to gain muscle’. ‘GA’ commented: ‘Causing increases in serotonin can reduce dopamine and this decreases muscle contractions, this can have a negative effect on muscle building […]’. ‘M’ commented: ‘I have reviewed some evidence that suggest SSRIs can affect the HPA and/or HTP [HPT] axes [referring to the hypothalamic–pituitary–adrenal axis and the hypothalamic–pituitary–thyroid axis] and this may be responsible for causing low testosterone levels’.

Converse to the above explanations, some other posters suggested functional reasoning behind decreased muscle mass – linked to reductions in ability to exercise brought on by SSRI use. However, this was a minority of posters, and clashed with the claims of others who suggested that exercise frequency and intensity was unchanged or increased, and that perceived reductions in lean muscle mass were reflective of beginning SSRI medications only, as opposed to later subsequent changes in exercise regime. However, some postings suggested the opposite of this. For example, ‘DC’ commented that while on SSRIs he had ‘zero drive or desire to go to the gym or eat like he used to’. Another poster: ‘MO’, commented more generally, suggesting: ‘some of these serotonin agent medications will eat-away-at your commitment discipline and aggression-levels to make you “happier”’. Another poster: ‘GAx’ commented: ‘There are loads of negative effects that SSRIs can make happen that render exercising more difficult’.

## Discussion

The prevalence of findings suggesting self-reported correlations between SSRI antidepressants and muscle loss, mainly in males, is notable, particularly due to the relatively small, and randomly selected sample. In reviewing a more in-depth Google analysis of indexed keywords, more forums not analysed within this study sample match similar keyword search criteria. This suggests that despite minimal current scholarly research exploring connections between SSRIs and reductions in lean muscle as a negative side-effect, a perceived and significant anecdotal connection exists in online forums, and this topic likely deserves further and structured scientific investigation.

Further study is important due to the negative physical effects anecdotally self-reported, and saliently how such effects may trigger compound impacts upon the mental health of such individuals, who have already sought treatment and for which medications have been prescribed. Such negative mental effects were discussed within this study’s sample. Notably, some SSRI class medications record muscle weakness as a possible side-effect.^27,28^ However, this is listed as temporary, and not explored as linked to lasting physical changes or significant, perceived bodily recomposition. Some postings analysed in this study suggested SSRIs may interfere with testosterone production, and suggest this may be a potential mechanism to explain rapidly reduced muscle mass, inability to build lean muscle in men, and increased body fat and changed muscle/fat distribution. Some posters highlighted such effects as beginning following SSRI treatment, and occurring despite self-reported regular weight training exercise (and possibly, as suggested by some posters) self-perceived adequate diet and rest. However, after cross-checking postings flagged as replying to such suggestions, some posters reported having their testosterone levels checked, and reported these as falling within ‘normal range’. Regardless, this does not concretely rule out a possible perceived correlation between SSRIs and testosterone reduction, as many men highlighted they are were not aware of their ‘baseline’ testosterone reading prior to beginning SSRI treatment (and indeed these claims of *being tested*, are unverifiable). This is a key limitation of anecdotal reporting of side-effects, and one well discussed at length in relevant research.^9–12^ Despite this, some recent research *has* explored linkages between SSRIs; antidepressants and testosterone.^26,29–31^ However, researchers note challenges establishing direct SSRI-testosterone-reduction-correlations.^26,30,31^

Exploring existing evidence, there are several possible mechanisms by which SSRIs *may* contribute to reductions lean muscle mass. SSRIs are commonly prescribed antidepressants that influence both central and peripheral physiological processes, with this influence carrying potential implications over human muscle health. One key mechanism through which SSRIs may contribute to muscle loss is via their primary mechanism of increasing serotonin, a neurotransmitter for which elevated levels can affect both the brain and skeletal muscle. The central fatigue hypothesis posits that increased serotonin activity in the brain leads to fatigue, reduced motor drive, and lower levels of physical activity, all of which can contribute to muscle atrophy.^32–36^ SSRIs can also impact the hypothalamic–pituitary–adrenal axis, which plays a central role in regulating cortisol, a hormone involved in stress responses.^37–40^ Elevated waking cortisol levels, which some have suggested may result from SSRI use,^40,41^ could induce muscle catabolism, cortisol correlated negatively with lean muscle mass.^42^ Thus, SSRIs *could* impair the energy available for muscle repair and regeneration, potentially leading to muscle degradation.^40–42^ Such proposed mechanisms suggest that SSRI treatment *may* be able to contribute to muscle loss, warranting further investigation into the long-term effects of antidepressant therapy on musculoskeletal health. Speaking to these points, a recent study by Andersson et al., published in *Obesity* in October 2024^43^ examined the impact of antidepressant use on body composition and cardiometabolic risks. The researchers analysed magnetic resonance imaging (MRI) data from 40,174 participants in the UK Biobank, comparing individuals using SSRIs and tricyclic antidepressants (TCAs) with matched controls. Results revealed that that ‘SSRI users had more visceral fat, smaller muscle volume, and higher muscle fat infiltration compared with matched control individuals’ (p. 1). In particular, male SSRI users were found to have an elevated risk of cardiovascular disease (CVD), and an unhealthier body composition profile. Both male and female TCA users exhibited decreased muscle volume and a heightened risk of developing type 2 diabetes. Notably, adverse changes in both male and female participants’ body composition were not reflected by body weight or body mass index (BMI) measurements, highlighting the importance of using more detailed assessments when monitoring individuals on antidepressant treatment.

Further to the above considerations, concerns of perceived reductions in lean muscle mass noted within forum poster discussions are also problematic from a societal standpoint. Numerous recent sociological research has demonstrated that pressures regarding ‘ideal’, ‘acceptable’, and ‘desirable’ bodily presentations for persons of all genders are linked to mental health concerns in the UK population.^44–51^ Such concerns and prevalence for ill-effects are only increasing with the popularity of various social media platforms.^44,45,47^ In tandem, an emerging body of literature suggests SSRIs are commonly prescribed to young people suffering body dysmorphia, low bodily self-esteem, and numerous other considerations that intertwine the psychological with the physical.^50,51^ If such medications can negatively impact physicality; physical appearance as a side-effect, then this may cause immediate and special harm to the populations described above. More scientific and sociological research is required to examine any possible correlations, and determine presence and extent of any effects. If such a correlation can be established, it is reasonable to suggest prescribing guidelines for the above demographic should be revisited, to avoid (unintentionally) causing harm through the prescription of medications unsuitable for such comorbidities.

## Acknowledgement of study limitations

While the study provides insight into a little-explored phenomenon, several limitations must be acknowledged. The voluntary nature of forum participation may introduce self-selection bias; with the dataset potentially overrepresenting individuals who have strong concerns about SSRI-related muscle changes, or indeed, are experiencing these to the degree that they felt compelled to seek help and record their experiences in online forums. Furthermore, as forum posts are inherently anonymous, verifying the accuracy of self-reported experiences was not possible. Thus, this is a study *of what people share happened to them,* in online spaces, rather than a study of verifiable accounts of *what was actually experienced by people*. Further, while 202 postings were sampled, not all of these captured postings contained data that could be linked to specific antidepressants (e.g. SSRI class medications). Similarly, demographic details such as age were not always available, and the availability of this information would have strengthened analysis. Additionally, it would be desirable to include additional illness-focussed contextual information regarding the experiences of various forum posters – for example, whether posters we experiencing depression, or were in remission at the time of posting. However, this information was not available in almost all cases. Despite these constraints, the findings highlight an important gap in existing research and underscore the need for further clinical investigation into potential physiological effects of SSRI medications on human muscle composition, growth, and function.

## Conclusions

This research has presented an investigation of some online and anecdotal ‘real world’ accounts that suggest SSRI antidepressants are perceived by some to reduce lean muscle mass, mostly in men, as a possible medication side-effect. Only one existing study reports this to any extent (Andersson et al., 2024), beyond sporadic mentions of temporary muscle weakness. However, discussions of this possible side-effect and the prevalence of findings suggest a need for further scientific enquiry. Investigations are necessary to grow knowledge on possible correlations, widen academic conversation, and ultimately to safeguard against any possible psychological and physical harm to already vulnerable individuals seeking help for conditions for which SSRI antidepressant medications are routinely prescribed.

## Supplemental Material

Supplemental Material - SSRI antidepressants and perceived loss of lean muscle in men: A qualitative exploration of some online anecdotal concerns Supplemental Material for SSRI antidepressants and perceived loss of lean muscle in men: A qualitative exploration of some online anecdotal concerns by Nicholas Norman Adams in International Journal of Risk & Safety in Medicine.

## Note

1. It should be noted that all poster initials used in this publication are pseudonyms.
