# Supplementary material for: SSRI antidepressants and perceived loss of lean muscle in men: A qualitative exploration of some online anecdotal concerns
Source: Int J Risk Saf Med. 2025 May 23;36(4):208–17. doi: 10.1177/09246479251346445 (PMC12552761; doi:10.1177/09246479251346445)
Supplement: Supplemental Material - SSRI antidepressants and perceived loss of lean muscle in men: A qualitative exploration of some online anecdotal concerns [file sj-pdf-1-jrk-10.1177_09246479251346445.pdf]

**Reviews for "SSRI antidepressants and perceived loss of lean muscle in men: a qualitative exploration of some online anecdotal concerns"**
**Authors:** Nicholas Norman Adams
**DOI**:

## Original Submission

## Reviewer #1

**Reviewer Recommendation Term:** Revise and resubmit pending major revisions

| | |
|---|---|
| Are you willing to review the revision of this manuscript? | Yes |
| Originality, novelty and significance of results: | Good |
| Technical Quality of Work: | Adequate |
| Comprehensibility and Presentation of Paper: | Adequate |
| What is the overall impression: | Adequate |

| | | |
|---|---|---|
| Is the article significant to the field? | [1-5] | 3 |
| Is the article appropriate for the journal? | [1-5] | 3 |
| Is the work an original contribution? | [1-5] | 4 |
| Are the conclusions adequately supported by the data? | [1-5] | 2 |
| Is the research interesting and important? | [1-5] | 4 |
| Is the level of English adequate? | [1-5] | 4 |

**Narrative (as sent to corresponding author):**

Thank you for the opportunity to review this manuscript. The author has highlighted a less-known side effect of SSRI and has used appropriate methods for such an initial step. Below are few comments:

Major comments:

1. The author may reframe methods and results as per qualitative study reporting guidelines - please see what suits the best: https://www.equator-network.org/reporting-guidelines-study-design/qualitative-research/

2. The author may attempt to review physiological processes that may contribute to lean muscle loss with SSRI - the current manuscript lacks neurophysiological evidence

3. The author must evaluate the verbatim critically and objectively assess the claims of the verbatim statements; this will provide more reliability on the claims made about muscle

mass loss. Further, the author must present all the views of the person regarding SSRI whose verbatim is used. For example:

For example, one male: 'R'1 spoke of beginning an SSRI medication (medication name not specified) to treat depression and anxiety. After a short spell on medication, his mood began to lift. However, he noticed improvements coincided with a shift in body composition, describing a perceived rapid loss of muscle mass and a noticeable increase in body fat, with loss of definition in all muscles (most notable in the stomach, chest, and arms), and the self-identified onset of gynecomastia:

"I noticed that the make-up of my body began to change quickly. I began to shed my naturally lean muscle and I grew a layer of fat, which gave me a 'man boob' look. To me it seemed like all the muscle in my body got soft, significantly, within a matter of weeks"

What else does R say about the other themes identified? In such a study, the views of each individual should be analysed in all the themes identified - this will give a holistic picture of the person's overall assessment capacity. Only when we know what R feels about all the themes does the claim of side effects become more reliable.

Minor comment:

1. It is perhaps better if the author sticks to the findings of the study and does not interpolate further views - this may be conjectural

2. The author may attempt to clarify the timing of these verbatims - were the participants out of depression? Symptoms of depression can also modulate these responses.

## Reviewer #2

**Reviewer Recommendation Term**: Revise and resubmit pending major revisions

| | |
|---|---|
| Are you willing to review the revision of this manuscript? | No: Not enough time and this is one the editor can decide on |
| Originality, novelty and significance of results: | Adequate |
| Technical Quality of Work: | Adequate |
| Comprehensibility and Presentation of Paper: | Adequate |
| What is the overall impression: | Good |

| | | |
|---|---|---|
| Is the article significant to the field? | [1-5] | 3 |
| Is the article appropriate for the journal? | [1-5] | 3 |
| Is the work an original contribution? | [1-5] | 3 |
| Are the conclusions adequately supported by the data? | [1-5] | 3 |

| | | |
|---|---|---|
| Is the research interesting and important? | [1-5] | 3 |
| Is the level of English adequate? | [1-5] | 3 |

**Narrative (as sent to corresponding author):**

1. There is some duplication in the Keywords. I would suggest changing to: SSRI antidepressants; SSRI side-effects; lean muscle loss; social pharmacy approaches.

2. The second paragraph of the Introduction says: "… particularly with regards to gathering qualitative data exploring patient's lived experiences of antidepressant use and side-effects."

It should be patients' instead of patient's (ie. the lived experiences of multiple patients).

3. In the first paragraph of the Methods section, I would suggest adding the word "and": "Study began with a linked Google (advanced) keyword search for "antidepressants", "muscle loss", [and] "side-effects"."

4. There is arguably overuse of em dashes which makes the text jarring to read in a few places. Some of these could possibly be replaced with commas or reworded slightly.

For example: "A final—additional—level of coding was used to link descriptive comments to specific types of antidepressant—where this information was available."

It could perhaps be changed to "A final level of coding was used to link descriptive comments to specific types of antidepressant where this information was available."

5. In the text, the references jump from 21 to 37.

6. The section titled "Theme 3: SSRIs and 'loss of lean muscle mass'" says:

"'C' commented that he was unable to find much info about this "on the internet" and asked other forum posters whether SSRIs were the cause, if other information was available, and whether there were alternative medications without these effects."

Given that the word "info" isn't part of a direct quote, I would suggest writing it more formally as "information".

7. The third paragraph of the Discussion section says: "While some suggest SSRIs may reduce testosterone levels, this reduction is not always statistically significant and may be attributed to other factors, for example, depression and low mood for which medications are prescribed to combat."

There is no real evidence that depression or low mood can reduce testosterone. This claim is unsubstantiated and unreferenced.

8. The Introduction, Discussion and Conclusions sections all note that there is no existing literature or clinical study reporting a link between SSRIs and muscle loss. However, there is in fact such a study recently published:

Andersson P, Linge J, Gurholt TP, Sønderby IE, Hindley G, Andreassen OA, Dahlqvist Leinhard O. Poor muscle health and cardiometabolic risks associated with antidepressant treatment. Obesity (Silver Spring). 2024 Oct;32(10):1857-1869. doi: 10.1002/oby.24085. PMID: 39315407.

It reported that "SSRI users had more visceral fat, smaller muscle volume, and higher muscle fat infiltration compared with matched control individuals."

The Andersson article may not have been available while the current manuscript was being written. However, it's an important article to include if possible, as it shows that the forum observations are indeed supported in the literature.

Summary: The article reports on a novel and potentially serious complication of SSRI treatment. I would recommend that the author be given the opportunity to review and address the above points before accepting for publication.

## Author’s reply to the reviews:

**Reviewer #1:**

Thank you for the opportunity to review this manuscript. The author has highlighted a less-known side effect of SSRI and has used appropriate methods for such an initial step. Below are a few comments:

Very many thanks for your helpful comments. I have addressed all of these below. I have highlighted these responses in yellow for clarity. Thank you again, this has resulted in a stronger manuscript. Very best. Major comments: 1. The author may reframe methods and results as per qualitative study reporting guidelines - please see what suits the best: https://eur01.safelinks.protection.outlook.com/?url=https%3A%2F%2Fwww.equator-network.org%2Freporting-guidelines-study-design%2Fqualitative-research%2F&data=05%7C02%7Cn.adams5%40rgu.ac.uk%7C307708359b5c489e487a08dd70804ed9%7C51a0a69c0e4f4b3db64212e013198635%7C0%7C0%7C638790418582715983%7CUnknown%7CTWFpbGZsb3d8eyJFbXB0eU1hcGkiOnRydWUsIlYiOiIwLjAuMDAwMCIsIlAiOiJXaW4zMiIsIkFOIjoiTWFpbCIsIldUIjoyfQ%3D%3D%7C0%7C%7C%7C&sdata=itb2Yf2%2BkMxw7HEkPWVPZN1fyfh8eN7bAfesmjLMTg0%3D&reserved=0

Thank you, the Standards for Reporting Qualitative Research (SRQR) have been followed and all sections have been cross-checked and edited to make sure they align with these.

2. The author may attempt to review physiological processes that may contribute to lean muscle loss with SSRI - the current manuscript lacks neurophysiological evidence

Thank you for this comments, I’ve now added a section on this, and this has strengthened the overall narrative and focus of the work – many thanks. 3. The author must evaluate the verbatim critically and objectively assess the claims of the verbatim statements; this will provide more reliability on the claims made about muscle mass loss. Further, the author must present all the views of the person regarding SSRI whose verbatim is used. For example: For example, one male: 'R'1 spoke of beginning an SSRI medication (medication name not specified) to treat depression and anxiety. After a short spell on medication, his mood began to lift. However, he noticed improvements coincided with a shift in body composition, describing a perceived rapid loss of muscle mass and a noticeable increase in body fat, with loss of definition in all muscles (most notable in the stomach, chest, and arms), and the self-identified onset of gynecomastia: "I noticed that the make-up of my body began to change quickly. I began to shed my naturally lean muscle and I grew a layer of fat, which gave me a 'man boob' look. To me it seemed like all the muscle in my body got soft, significantly, within a matter of weeks"

What else does R say about the other themes identified? In such a study, the views of each individual should be analysed in all the themes identified - this will give a holistic picture of the person's overall assessment capacity. Only when we know what R feels about all the themes does the claim of side effects become more reliable.

Thank you for these comments. This is challenging, as it is not the focus of this work to claim to generate reliability through evaluation of the claims of these statements (as they are subjective perceptions), this would contravene the qualitative principles of the research (and indeed the sociological reporting of participants experiences). Instead, I've added a section openly highlighting the lack of verifiability regarding what people say in online spaces, vs. what may have occurred in 'real life'. This positions the work as inherently unverifiable (as with much qualitative research), but valuable from the perspective of suggesting future structured research is required. Thank you again for your comments here. Minor comments: 1. It is perhaps better if the author sticks to the findings of the study and does not interpolate further views - this may be conjectural

Thanks for this comment, I've completed an edit and removed / softened sections that may have been interpreted as more speculative.

2. The author may attempt to clarify the timing of these verbatims - were the participants out of depression? Symptoms of depression can also modulate these responses.

It's not possible to know this – as this was not mentioned my participants (but where inferred this has been spoken to). I an cautious not to over represent the data with variables I do not have – this is often the conundrum of qualitative data—and even more so with Netnographic (online collected) data. I've spoken to this within the work and added a section in limitations re this.

Thank you again for all of your helpful and constructive comments on this manuscript.

**Reviewer #2:**

1. There is some duplication in the Keywords. I would suggest changing to: SSRI antidepressants; SSRI side-effects; lean muscle loss; social pharmacy approaches.

Thanks, this has now been changed. 2. The second paragraph of the Introduction says: "… particularly with regards to gathering qualitative data exploring patient's lived experiences of antidepressant use and side-effects." It should be patients' instead of patient's (ie. the lived experiences of multiple patients).

Thanks, this has now been changed. 3. In the first paragraph of the Methods section, I would suggest adding the word "and": "Study began with a linked Google (advanced) keyword search for "antidepressants", "muscle loss", [and] "side-effects"."

Thanks – this has been changed. 4. There is arguably overuse of em dashes which makes the text jarring to read in a few places. Some of these could possibly be replaced with commas or reworded slightly. For example: "A final—additional—level of coding was used to link descriptive comments to specific types of antidepressant—where this information was available." It could perhaps be changed to "A final level of coding was used to link descriptive comments to specific types of antidepressant where this information was available."

This is a good point, I've amended these to reduce the volume of dashes.

5. In the text, the references jump from 21 to 37.

Thanks, this has now been corrected. 6. The section titled "Theme 3: SSRIs and 'loss of lean muscle mass'" says: "'C' commented that he was unable to find much info about this "on the internet" and asked other forum posters whether SSRIs were the cause, if other information was available, and whether there were alternative medications without these effects." Given that the word "info" isn't part of a direct quote, I would suggest writing it more formally as "information".

Thanks for highlighting this and I absolutely agree, this has now been changed. 7. The third paragraph of the Discussion section says: "While some suggest SSRIs may reduce testosterone levels, this reduction is not always statistically significant and may be attributed to other factors, for example, depression and low mood for which medications are prescribed to combat." There is no real evidence that depression or low mood can reduce testosterone. This claim is unsubstantiated and unreferenced.

Thanks for highlighting this. There is a reference in the ref list (that should have been highlighted here, but ultimately, the study is not conclusive, so I have softened this claim, reworded and agree with what you have said re no real evidence currently existing. 8. The Introduction, Discussion and Conclusions sections all note that there is no existing literature or clinical study reporting a link between SSRIs and muscle loss. However, there is in fact such a study recently published: Andersson P, Linge J, Gurholt TP, Sønderby IE, Hindley G, Andreassen OA, Dahlqvist Leinhard O. Poor muscle health and cardiometabolic risks associated with antidepressant treatment. Obesity (Silver Spring). 2024 Oct;32(10):1857-

1869. doi: 10.1002/oby.24085. PMID: 39315407. It reported that "SSRI users had more visceral fat, smaller muscle volume, and higher muscle fat infiltration compared with matched control individuals." The Andersson article may not have been available while the current manuscript was being written. However, it's an important article to include if possible, as it shows that the forum observations are indeed supported in the literature.

This is great. No, it wasn’t available, I’ve now included this and spoken to its findings within the work. Thank you for highlighting this a very interesting and relevant study. Summary: The article reports on a novel and potentially serious complication of SSRI treatment. I would recommend that the author be given the opportunity to review and address the above points before accepting for publication.

Thank you once again for your helpful and constructive comments. Very best.

## Reviewers' response to the revision 1:

## Reviewer #1

**Reviewer Recommendation Term**: Accept as is

| | |
|---|---|
| Are you willing to review the revision of this manuscript? | Yes |
| Originality, novelty and significance of results: | Good |
| Technical Quality of Work: | Good |
| Comprehensibility and Presentation of Paper: | Good |
| What is the overall impression: | Good |

| | | |
|---|---|---|
| Is the article significant to the field? | [1-5] | 3 |
| Is the article appropriate for the journal? | [1-5] | 3 |
| Is the work an original contribution? | [1-5] | 4 |
| Are the conclusions adequately supported by the data? | [1-5] | 2 |
| Is the research interesting and important? | [1-5] | 4 |
| Is the level of English adequate? | [1-5] | 4 |

**Narrative (as sent to corresponding author):**

Thank you - the author has responded to the suggestions adequately. I do not have any further comments. All the best!

## Reviewer #2

**Reviewer Recommendation Term**: Accept as is

| | |
|---|---|
| Are you willing to review the revision of this manuscript? | No: Editors have to make decisions re accepting not reviewers |
| Originality, novelty and significance of results: | Excellent |
| Technical Quality of Work: | Good |
| Comprehensibility and Presentation of Paper: | Good |
| What is the overall impression: | Good |

| | | |
|---|---|---|
| Is the article significant to the field? | [1-5] | 3 |
| Is the article appropriate for the journal? | [1-5] | 3 |
| Is the work an original contribution? | [1-5] | 3 |
| Are the conclusions adequately supported by the data? | [1-5] | 3 |
| Is the research interesting and important? | [1-5] | 3 |
| Is the level of English adequate? | [1-5] | 3 |

**Narrative (as sent to corresponding author):**

1. Is the paper logical with a concise ordering of ideas?

Yes

2. Does the paper describe sound research methods, analysis & interpretation? Are limitations to the study included and discussed?

Yes

3. In case the authors used established methodology, are the results of all previous studies been searched for and presented in a concise form? Has the current work had impact on the local practice and policies?

Yes

4. Is the paper well referenced and using the Vancouver format? Are the majority of references within the last 3-5 years? Main findings should be traced back to their origin.

fine

5. Is the paper consistent with the purpose and scope of JRS? Does the paper discuss risk AND benefit?

yes

6. What is the quality of the readability of the paper in English? Is the presentation/layout clear? Have the author guidelines been followed?

Good

7. Are the ideas in the paper original and the significance of the research described? Are the results of likely interest to an international audience?

Yes

8. Is the content timely?

Yes

9. Does the paper build on and advance the knowledge of papers published in JRS and other international journals? If the paper describes methodology and results of original / experimental clinical (health) research, does it build on the existing knowledge, coming from systematic reviews? Have the searches for the rationale been rigorously done?

Yes

**THE EDITOR-IN-CHIEF DECIDED TO ACCEPT THE PAPER**
